# The impact of individual differences on jurors’ note taking during trials and recall of trial evidence, and the association between the type of evidence recalled and verdicts

**DOI:** 10.1371/journal.pone.0212491

**Published:** 2019-02-19

**Authors:** Joanna Lorek, Luna C. M. Centifanti, Minna Lyons, Craig Thorley

**Affiliations:** 1 Department of Psychological Sciences, University of Liverpool, Liverpool, United Kingdom; 2 Department of Psychology, James Cook University, Townsville, Australia; University of St Andrews, UNITED KINGDOM

## Abstract

Although note taking during trials is known to enhance jurors’ recall of trial evidence, little is known about whether individual differences in note taking underpin this effect. Individual differences in handwriting speed, working memory, and attention may influence juror’s note taking. This, in turn, may influence their recall. It may also be the case that if jurors note down and recall more incriminating than non-incriminating evidence (or vice versa), then this may predict their verdict. Three studies examined the associations between the aforementioned individual differences, the amount of critical evidence jurors noted down during a trial, the amount of critical evidence they recalled, and the verdicts they reached. Participants had their handwriting speed, short-term memory, working memory, and attention assessed. They then watched a trial video (some took notes), reached a verdict, and recalled as much trial information as possible. We found that jurors with faster handwriting speed (Study 1), higher short-term memory capacity (Study 2), and higher sustained attention capacity (Study 3) noted down, and later recalled, the most critical trial evidence. However, working memory storage capacity, information processing ability (Study 2) and divided attention (Study 3) were not associated with note taking or recall. Further, the type of critical evidence jurors predominantly recalled predicted their verdicts, such that jurors who recalled more incriminating evidence were more likely to reach a guilty verdict, and jurors who recalled more non-incriminating evidence were less likely to do so. The implications of these findings are discussed.

## Introduction

During trials, jurors need to encode the evidence, legal arguments, and judicial instructions presented to them, store these in memory throughout the trial, and recall them during deliberation to reach a fair verdict. Jurors’ recall of trial evidence, however, has been found to be incomplete and inaccurate [1–3]. These memory failures can influence verdicts, such that jurors who forget incriminating evidence are less likely to find a defendant guilty than those who remember it [4]. Note taking during trials has been shown to be an effective memory aid [1,3,5,6], with jurors recalling a similar amount of trial information irrespective of whether they can or cannot access their notes during a memory test [7,8]. It may be the act of note taking that enhances encoding of trial information and this improves later recall of that information and thus, jurors get no further benefit from accessing their notes during the memory test. Researchers have yet to examine the impact of individual differences on note taking during trials, but a small number of studies in the educational psychology literature have examined whether individual differences can predict the volume of notes students’ take during lectures and the volume of lecture material they can later recall [9,10]. Here, we examined whether individual differences in handwriting speed, memory, and attention can predict jurors’ note taking during trials and their recall of trial evidence. Further, it may be that the type of trial evidence jurors predominantly recall can statistically predict their verdicts. We therefore also examined this possibility.

### Handwriting speed

In educational psychology research, undergraduate students with faster handwriting speed make better quality notes, as measured by completeness of explanations for each topic mentioned during a lecture [11]. Moreover, the quality of their notes is positively associated with the quality of their recall of the lecture material [9,10]. Note takers with a slower handwriting speed may find it difficult to note down the lecture information before it is forgotten and this may place a load on their cognitive resources, such as working memory, which are necessary to process incoming information [12,13]. Thus, fast handwriting speed has the potential to decrease the weight placed on the limited resources of working memory; this would be especially important for jurors, such that individual differences in handwriting speed may be associated with their recall. Given the findings from educational psychology, jurors’ handwriting speed may play a central role in determining how many notes they make during a trial and how much trial information they can later recall. It may be that jurors with faster handwriting speed have an advantage over those with slower handwriting speed as they can take more notes during a trial.

### Memory and information processing

Note taking is cognitively effortful as it involves temporary storing and manipulating information in memory [13]. Thus, individual differences in the cognitive processes of temporary storing, manipulating, and processing new information may influence note taking skills. However, the evidence from educational psychology provides mixed results. Bui and Myerson [14] summarise the previous studies that have examined the association between short-term memory capacity, working memory capacity, information processing ability, and note taking. Before we review the relevant literature, we briefly define short-term and working memory.

Working memory refers to the storage, manipulation and updating of incoming information [15,16]. It is a limited capacity system where the phonological loop and the visuospatial sketchpad are responsible for the storage function, and the central executive is responsible for manipulating and updating information [17]. Short-term memory could be considered a subcomponent of working memory. Short-term memory is responsible for the storage of new information [18] which is equivalent to the role of the phonological loop and the visuospatial sketchpad. However, short-term memory does not involve any manipulation or processing of information. One key distinction between working memory and short-term memory is that in order to manipulate the incoming information, working memory uses more attentional resources [17]. Typically, working memory capacity is assessed using complex span tasks (such as a reading span task) whereas short-term memory capacity is assessed using more traditional span tasks (such as a letter span task) which measure the storage component of working memory.

To our best knowledge, no study has explored the relationship between note taking and short-term memory capacity by using traditional short-term memory span tasks. Thus, we used a letter span task to examine whether jurors’ short-term memory capacity was associated with the amount of notes they made during a trial, and whether it was also indirectly associated with the amount of trial evidence they subsequently recalled.

Furthermore, complex span tasks (such as the listening span task) have been used to assess working memory storage capacity. No association has been found between students’ working memory storage capacity and the amount of lecture ideas recorded in notes [10]. However, in Peverly, Garner and Vekaria’s study [10], the association approached significance (p = 0.08) and they argue the non-significant results may have been significant with a larger sample size (due to a lack of statistical power). Therefore, we used a larger sample size and examined whether an association can be found in a different context (i.e. during trials). Others have found a significant association between working memory and lecture note taking when students were asked to organise their notes [19]. We used the listening span task to investigate whether jurors’ working memory capacity was associated with the amount of notes they took during a trial, and whether it was also indirectly associated with the amount of trial evidence they subsequently recalled.

Lastly, information processing tasks (such as the word reordering task) have been used to assess the cognitive ability to process/manipulate information. Students’ scores on the word reordering task have been found to predict the number of propositions and main ideas they note down during a lecture [20]. The number of propositions and main ideas students noted down also predicted their performance on a course exam. Thus, students with lower information processing ability take less effective lecture notes and subsequently recall less lecture material. It should be noted that throughout the word re-ordering task, our participants were able to see the words and did not need to store them in working memory. Although the task does not require participants to hold the information in memory, it does measure the cognitive function of information processing which is closely related to the cognitive processes of manipulating information in the central executive. Therefore, we used the word reordering task to explore whether jurors’ information processing ability was associated with the amount of notes they took during a trial, and whether it was also indirectly associated with the amount of trial evidence they subsequently recalled.

### Attention

During trials, it is crucial that jurors stay attentive and focus on the evidence presented as they need to encode as much information as possible to be able to remember it when reaching verdicts. Trials vary in length and can last from a few hours to a few months. Thus, jurors’ ability to use their limited cognitive resources [13] over long periods of time (sustained attention) and their ability to split attention between note taking and listening to trial proceedings (divided attention) may be associated with their note taking during trials, which may subsequently be associated with their recall of trial information.

In the educational psychology literature, sustained attention has been found to be positively related to the amount of notes taken during a lecture [10]. Furthermore, the amount of notes taken predicts the amount of lecture information recalled. Similar findings have been obtained in other studies [21,22]. Considering the lengthy proceedings of trials, sustained attention may be an important factor affecting jurors’ note taking during trials and their recall of trial information. It is possible that the findings from studies with undergraduate students apply to jurors. According to the overload theory, attentional resources become exhausted when attempting to maintain focus on the same information for extended periods of time [23,24]. Thus, jurors with lower levels of sustained attention capacity (relative to those with higher sustained attention capacity) may be least likely to maintain focus on a trial over extended periods of time. This could then reduce the number of notes they take and result in them recalling less trial information.

It is currently unknown whether note taking can act as a form of divided attention that is detrimental to encoding of new information for those with low divided attention capacity. In the wider memory literature, the impact of divided attention on memory of simple material (e.g., words), has been studied extensively using a dual-task paradigm. Typically, participants are required to study word lists under a full attention condition or a divided attention condition (i.e., whilst performing a secondary task that diverts attention from the word lists). Divided attention, when compared to full attention, during encoding generally decreases participants’ free recall, cued recall, and recognition of words lists [25–28].

We acknowledge that the aforementioned studies examined divided attention using tasks that typically divert attention away from the to-be-learned information during encoding which is detrimental to memory. However, note taking typically focuses jurors’ attention on the to-be-learned information at encoding which may benefit memory. It is, however, reasonable to question whether jurors with lower divided attention capacity will find it harder to divide attention between taking notes and encoding the trial content, which could have a negative impact on their note taking and subsequent recall of trial information.

### Critical evidence and verdicts

Jurors’ recall of trial evidence can be incomplete. For instance, Costabile and Klein [4] found that just over one-fifth of mock jurors forgot about the defendant’s wire-tapped confession to the murder. Importantly, those jurors who forgot about the wire-tapped confession were less likely to find the defendant guilty. Thus, there is a link between recall of trial information and verdicts.

Irrespective of whether or not jurors take notes, the type of critical evidence they recall (i.e. the most important pieces of incriminating/non-incriminating trial evidence that could influence a juror’s verdict) may predict their verdicts. Whilst reaching verdicts, jurors may remember more incriminating (or non-incriminating) trial evidence and may then find a defendant guilty (not guilty) because of this. To date, no study has examined whether a bias towards recalling more incriminating or non-incriminating evidence can predict jurors’ verdicts. Therefore, we assessed the association between the type of critical evidence jurors predominantly recalled and the verdicts they reached.

### Aims

The purpose of the three studies was to investigate (1) the impact of individual differences on juror note taking and recall of critical trial evidence, and (2) the impact of the type of trial evidence recalled on verdicts. In relation to the first of these, Study 1 focused on individual differences in handwriting speed, Study 2 focused on individual differences in short-term memory, working memory, and information processing ability, and Study 3 focused on individual differences in divided and sustained attention.

## Study 1

The principal aims of Study 1 were to investigate the associations between mock jurors’ handwriting speed, the amount of critical evidence they noted down during a trial, the amount of critical evidence they recalled, and whether the type of evidence recalled predicts their verdicts. We measured handwriting speed before the jurors watched a trial video (two-thirds of jurors were allowed to take notes whilst watching the video). Then all jurors reached a verdict and recalled as much trial information as possible. The non-note takers were our control group which helped us determine whether note taking enhances recall of trial information, as has been found previously [3,8].

We used a mediation model to test our novel hypothesis regarding whether handwriting speed would be associated with the amount of critical evidence jurors recalled, via the amount of critical evidence they noted down during the trial. It was expected that jurors with faster handwriting speed would note down the most critical evidence during the trial, which would increase the amount of critical evidence they recalled, in line with findings from educational psychology [10]. Furthermore, we hypothesised that jurors with faster handwriting speed would be able to recollect more trial evidence, and this would be through the greater amount of evidence they recorded in their notes during the trial. However, we did not expect a direct effect of handwriting speed on recall. This is because the recall test was self-paced, meaning jurors could take as much time as they wanted to write down what they remembered.

Of importance to juror decision making, we tested whether the type of critical evidence (incriminating or non-incriminating) that jurors predominantly recall predicts their verdicts. That is, the analysis assessed whether jurors who recalled more incriminating evidence were more likely to reach a guilty verdict, and whether those who recalled more non-incriminating evidence were more likely to reach a not guilty verdict. In line with previous studies [8], it was hypothesised that the note taking condition jurors are in (note taking vs. non-note taking) will not predict their verdict.

### Method

#### Participants and design

One hundred forty-one participants acted as mock jurors (henceforth called ‘jurors’) (24 males, 117 females). All were between 18 and 66 years of age (*M* = 20.33, *SD* = 6.26). Participants were drawn from undergraduate student sample and staff at a northwest English university. All met the eligibility criteria for jury service in England and Wales (i.e. they were between 18 and 75 years of age, on the electoral register, and lived in the UK for a period of at least 5 years since the age of 13). Prior to testing, all participants provided written consent confirming they wished to take part in the study. All received a payment in the form of course credit or a £10 voucher (based on if they were a student or staff, respectively). The study was approved by the Health and Life Sciences Committee on Research Ethics of the University of Liverpool.

Participants were assigned to one of three conditions: note taking without access to notes during the memory test (N = 60), note taking with access to notes during the memory test (N = 54) and no note taking (N = 27). Previous findings [7,8] have shown that jurors who either can or cannot access their notes during a memory test recall a similar amount of trial information. We investigated this further in the current study.

Participants were allocated to conditions in a quasi-random method, whereby testing for each condition took place on fixed days of the week. For instance, if participants completed the study on a Monday they were allocated to a non-note taking condition, whereas if participants took part in the study on a Tuesday they were in the note taking condition. Participants signed up for a session in groups of up to 3 without knowing what condition they would be in.

#### Stimuli

The Alphabet Fluency Task [29] was used to measure the jurors’ handwriting speed. When completing this test, participants were instructed to write the letters of the alphabet in a sequential order (from ‘‘a” to ‘‘z”) on a lined sheet of paper. The time limit for this was set to 45 seconds. Jurors commenced with lowercase letters and then switched to uppercase letters if they managed to write out the entire alphabet before the time elapsed. Each legibly written letter was given one point. A letter was considered legible if a researcher was able to identify it. Illegible letters were given zero points. The total number of points was tallied to form each participant's final score.

Jurors watched a 30-min video of a 1992 murder re-trial with the case name New Jersey vs. Daniel Bias. Trials are not broadcast in England and Wales, thus, footage from a US trial was used. In this trial, the defendant was accused of murdering his wife by shooting her in the head. The defendant claimed he is innocent and that his wife shot herself. The video was edited so that it contained the opening statements, the cross-examination of six witnesses and the defendant, the closing statements, and the judicial instructions. The verdict is not shown. Past research shows jurors are evenly split between guilty and not guilty verdicts for this trial [2,30–33] and that whether or not jurors take notes does not influence their verdicts [8].

Consistent with real trials in England and Wales [34], jurors were provided with blank lined notepads and pens for note taking. All jurors were also given a demographic/verdict questionnaire asking them their age, gender, and whether they considered the defendant to be guilty or not guilty. Finally, a 10-page A4 lined booklet was provided for the free recall test.

#### Procedure

Jurors were tested in a computer lab in groups of up to three. In each session, all participants were in the same condition. Each participant was seated at an individual PC. Throughout the study, they were unable to see each other performing the tasks due to desktop screen dividers. First, all jurors completed the Alphabet Fluency task. They were then informed that they would watch a trial video and once it was over they would reach a verdict and answer questions about the trial. No explicit reference was made to a memory test. They were then told whether or not they would be allowed to take notes during the trial. Note takers were then provided with a notepad and pen and the trial video commenced. After the video, they were asked to complete the demographic/verdict questionnaire. Next, all jurors completed the free recall test with no time limit. They were instructed to write down as much trial evidence as they could remember. Half of the note takers were allowed to access their notes throughout the memory test, the other half had their notes confiscated before the test. The participants were not informed prior to taking notes whether or not they would be allowed access to their notes during the memory test. In practice, we did not measure whether jurors looked at or used their notes during the memory test. Upon completion of the free recall test, all jurors were debriefed and the study ended. The study lasted approximately 60 mins.

#### Coding of notes and recall

The amount of critical evidence noted down and freely recalled was scored as follows. A coding sheet for critical evidence was based on the pilot study where participants identified the most important trial evidence. In the pilot study, thirty-three participants were asked to watch the trial video and then write down the ten most important pieces of trial evidence they believed could impact upon jurors’ verdicts. They were also asked to indicate whether each piece of evidence implied that the defendant was guilty or not guilty. Moreover, they were asked to rank these pieces of evidence from the most important to the least important. The pilot study used non-legal professionals (lay members of the public) to assess what they thought was the most important evidence presented during the trial. We believe this enables us to examine the evidence that potential jurors may feel is important to focus on when reaching verdicts. Combined, the participants identified 16 unique pieces of trial evidence that they believed could influence real jurors’ verdicts. Half of these implied that the defendant was guilty (henceforth called incriminating evidence) whereas the other half implied that he was not guilty (henceforth called non-incriminating evidence). For instance, the fact that the victim was right handed but was shot on left hand side of her head implies the defendant was guilty, so is an example of incriminating evidence. Conversely, the fact that the victim had previously threatened to kill herself implies that the defendant was not guilty, so is an example of non-incriminating evidence. In the present study, the lead author tallied the pieces of incriminating evidence (out of eight) and non-incriminating evidence (out of eight) noted down and freely recalled by participants.

All notes and free recall responses were also scored for the overall amount of correct information noted down and recalled, irrespective of whether it is critical evidence or not. A coding sheet containing all information that appeared in the trial video (taken from Thorley et al. [8]) was used to assess the amount of correct trial information participants noted down/recalled. A piece of trial information was classified as correct if it appeared in the trial and was correctly described. Any information that was repeated by more than one person during the trial was scored only once in the note taking and free recall results, irrespective of how many times this information was written down and whether or not it could be attributed to a specific source. Each correct piece of information noted down or recalled was awarded one point. These points were then tallied to provide jurors with single scores for the volume of correct pieces of trial information noted down and recalled. The amount of correct information noted down and recalled was used to check whether the current studies are in line with the previous findings where note taking during trials was found to improve recall of correct trial information.

Twenty percent of jurors’ notes and free recall responses were checked for inter-rater reliability by two independent raters who were blind to the aims of the study. One rater coded the notes and the other rater coded the free recall responses. The inter-rater agreement between the lead author and independent raters was 84% for notes and 85% for free recall. All disagreements were resolved by the lead author and an independent reviewer who compared them and determined the correct score.

### Results

Correlational analyses were used to examine the associations between handwriting speed, the volume of critical evidence noted down and recalled, and amount of correct information noted down and recalled (see Table 1).

**Table 1:** Descriptive statistics and zero-order correlations (Pearson’s *r*) showing jurors’ handwriting speed, the amount of critical evidence they noted down and recalled, and the overall amount of correct trial information they noted down and recalled.

Variable	Mean(±SD)	1	2	3	4
1. Handwriting Speed	72.42(±16.54)	-			
2. Critical Notes	8.17(±2.82)	.26**	-		
3. Critical Recall	6.28(±2.33)	.12	.37***	-	
4. Correct Notes	29.34(±15.19)	.32***	.72***	.23*	**-**
5. Correct Recall	19.01(±8.14)	.27**	.31***	.59***	.49***

* *p* < .05

** *p* < .01

*** *p* < .001

A simple mediation analysis was conducted to assess (1) the direct effect of handwriting speed on the amount of critical evidence recalled, and (2) the indirect effect of handwriting speed on recall through the amount of critical evidence noted down. The model included only those jurors who took notes during the trial.

**Fig 1:**
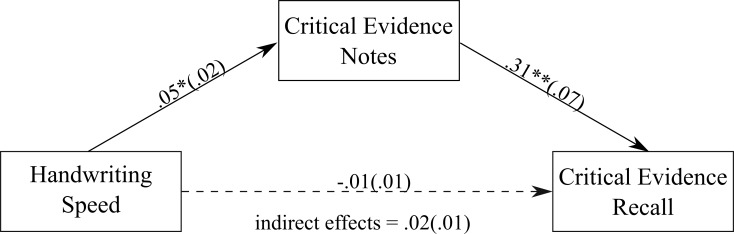
The mediation model showing the association between handwriting speed and the amount of critical evidence recalled, with the amount of critical evidence noted down as the mediator. Values on paths are unstandardised regression coefficients (SEs). * *p* < .01; ** *p* < .001.

All analyses were conducted using PROCESS (Version 2.16.1) for SPSS [35]. The results of these analyses are summarised in Fig 1. We presented unstandardised estimates, SE and 95% CI of the estimates. CI which do not include zero are significant. In addition, we reported κ^2^, which is an effect size for indirect effects in mediations [36], and *P*_*M*_, which is a ratio of the indirect effect to the total effect [37]. κ^2^ and *P*_*M*_ values were calculated using the MBESS package (version 4.4.3) [38] in R (version 3.4.3) [39].

Handwriting speed was found to be positively associated with the amount of critical evidence noted down during the trial (see Fig 1). There was also a positive association between the amount of critical evidence jurors noted down and the amount of critical evidence they recalled. Fig 1 shows a non-significant direct effect of handwriting speed on the amount of critical evidence recalled, unstandardised estimate = -.01 (SE = .01), 95% CI = -.04, .02. However, there was a significant indirect effect of handwriting speed on recall through the amount of critical evidence noted down during the trial, unstandardised estimate = .02 (SE = .01), 95% CI = .01, .03, κ^2^ = 0.11, *P*_*M*_ = 3.43 (κ^2^ indicates a medium effect size). More specifically, jurors with faster handwriting speed recalled a greater amount of critical evidence, via writing down the most critical evidence during the trial.

#### Incriminating/non-incriminating evidence and verdict

A logistic regression was conducted to assess whether the amount of incriminating evidence and the amount of non-incriminating evidence jurors recalled predicted their verdicts (0 = not guilty, 1 = guilty). The overall model significantly predicted the likelihood of jurors reaching a guilty verdict, correctly identifying 77.6% of cases (χ*^2^*(2) = 31.64, Cox & Snell *R^2^* = .31, Nagelkerke *R^2^* = .43, *p* < .001). The amount of incriminating evidence recalled statistically predicted jurors reaching a guilty verdict, B = .72 (SE = .22), Wald = 10.96, *p* = .001; OR = 2.05, 95% CI = 1.34, 3.14, such that for every additional piece of incriminating evidence recalled, jurors were 2.05 times more likely to reach a guilty verdict. Furthermore, the amount of non-incriminating evidence recalled negatively predicted jurors reaching a guilty verdict, B = -.75 (SE = .23), Wald = 10.91, *p* = .001; OR = 0.47, 95% CI = 0.30, 0.74, such that for every piece of non-incriminating evidence recalled, jurors were 2.13 times less likely to reach a guilty verdict.

#### Verdict

Seventy-three percent of jurors believed that the defendant was guilty: 78% in the access to notes during retrieval condition, 70% in the no access to notes condition, and 70% in the non-note taking condition. A logistic regression analysis was conducted to evaluate whether the condition that the jurors were in (note taking with access at retrieval, note taking with no access at retrieval, and non-note taking) was associated with their verdict. The regression revealed that the condition did not predict verdicts, (χ*^2^*(1) = .26, Cox & Snell *R*^2^ = .005, Nagelkerke *R*^2^ = .007, *p* = .40).

#### Benefits of note taking and note access at retrieval

An independent t-test was used to examine whether note-taking jurors recalled more correct trial information than non-note taking jurors. Note taking jurors recalled significantly more correct trial information (*M* = 20.07, *SD* = 7.94) than jurors who did not take notes (*M* = 14.56, *SD* = 7.60), *t*(139) = 3.27, *p* = .001 , *d* = 0.71.

We also examined whether note-taking jurors who could access their notes during the memory test recalled more correct trial information than note-taking jurors who did not have access to their notes during the memory test. There was no significant difference between the amount of trial information recalled by those who could access their notes during the memory test (*M* = 20.70, *SD* = 7.72) and those who could not (*M* = 19.50, *SD* = 8.15), *t*(112) = 0.81, *p* = .42, *d* = 0.15.

### Interim discussion

Study 1 is the first known study to show that jurors with faster handwriting speed recall a greater amount of critical evidence, and this is via being able to note down the most critical evidence during the trial. This finding is theoretically important as it shows that handwriting speed not only affects how much jurors can write down during a trial, but how much critical evidence they subsequently recall. In addition, Study 1 shows that jurors who recalled more incriminating evidence were more likely to find the defendant guilty whereas jurors who recalled more non-incriminating evidence were less likely to find the defendant guilty. This finding indicates that the type of trial evidence jurors predominantly recall is associated with the verdict they reach.

Our findings are in line with previous findings from the educational psychology literature, where the quality of notes mediated the association between handwriting speed and recall of lecture material [11]. We extend this by showing the amount of critical evidence noted down is a factor that mediates the association between note taking jurors’ handwriting speed and their recall of critical evidence. It may be that note takers with slower handwriting speed may find it difficult to note down the information before it is forgotten, which places a heavy cognitive load on their working memory [12,13]. Thus, individuals with fast handwriting speed may have more working memory resources available which they can utilise to take more notes. Thus, our second study investigated the role of working memory in jurors’ note taking and recall of trial evidence, and again considered whether the type of evidence recalled predicts jurors’ verdicts.

## Study 2

Study 2 examined the associations between jurors’ short-term memory capacity, working memory capacity, information processing ability, and the amount of critical evidence noted down/recalled. In addition, the study further explored the associations between the type of evidence jurors recalled and their verdicts. Presumably jurors who are able to store larger amounts of trial information in short-term memory will be able to take more notes and then recall more trial evidence. Thus, we examined the associations between short-term memory capacity, note taking and recall of trial information. Further, it can be presumed that those with higher levels of working memory storage capacity will be able to take more notes as they can store/manipulate larger amounts of incoming information in memory whilst focusing on making notes, and subsequently they will recall more trial evidence. However, findings from the educational psychology literature have provided conflicting results regarding the association between working memory storage capacity and lecture note taking [9,40] thus, we further explored this relationship. Furthermore, in line with research examining lecture note taking [20], we hypothesised that jurors with lower information processing ability would take fewer notes during the trial and subsequently recall less critical evidence. Lastly, we investigated whether the type of evidence (incriminating, non-incriminating) jurors recall predicts their verdicts. In Study 1, we demonstrated that jurors who recalled more incriminating evidence were more likely to reach a guilty verdict, whereas those who recalled more non-incriminating evidence were more likely to reach a not guilty verdict. We expected to find similar effects here.

### Method

#### Participants and design

Eighty-five jurors who were eligible for jury service in England and Wales (17 males, 68 females) between 18 and 61 years of age (*M* = 22.73, *SD* = 8.83) acted as mock jurors. Prior to testing, all participants provided written consent confirming they wished to take part in the study. As in Study 1, they were a combination of undergraduate students and staff at the first author’s university and received payment in the form of either course credit or a £10 voucher. They were assigned to one of two conditions: a note taking (N = 58) or a non-note taking condition (N = 27). Participants were assigned to conditions as in Study 1. The study was approved by the Health and Life Sciences Committee on Research Ethics of the University of Liverpool.

#### Stimuli

The Letter Span task is a measure of short-term memory storage capacity. This task involved verbally presenting jurors with lists of letters. Jurors were asked to remember the letters and verbally recall them in the same order as they were presented. None of the lists contained the same letter twice. On the first trial, the researcher read out a list of three letters (e.g., 'x, g, k') to each juror who was instructed to immediately repeat them back in the same order. If he/she repeated all letters correctly, they passed the trial and were given a list of four letters (e.g., 'w, b, o, l'). Each trial increased by one letter. If jurors failed at the first attempt (e.g., 6 letters), they were given a second attempt using the same number (e.g., 6) of a different set of letters. If they failed at the second attempt, the task was terminated. The length of the longest list a juror repeated correctly was his/her short-term memory capacity score.

The Listening Span task [41] is a measure of working memory storage capacity. Sixty sentences (between 11 and 16 words long) were pre-recorded. Half of the sentences did not make sense as they contained random words. There were five different levels which varied in the number of sentences presented from two to six. Each level consisted of three trials. For example, level one consisted of three trials of two sentences, level three consisted of three trials of three sentences, and so on. Jurors were required to complete two tasks. First, after each sentence was played, jurors were required to determine whether the sentence made sense by circling yes or no on an answer sheet. They were also required to remember the last word from each sentence for later recall. After each trial, jurors were prompted by a beep to recall the last word from each sentence in that trial and write them down in the same order as they had been presented. All jurors were given two practice trials before they began the task. The final score was the level at which jurors correctly recalled words on two out of three trials. If he/she scored one out of three on a trial he/she had half a point added to the final score. The final scores could range from zero to six.

The Word Reordering task [42] measures individual differences in information processing and information manipulation in working memory. Jurors were presented with six scrambled sentences, each consisting of ten words. The sentences were taken from a Health Psychology textbook. An example of a scrambled sentence would be “for norms men differently may social women be operating and”, and the solution would be “Social norms may be operating differently for men and women”. Jurors were instructed to reorganise the words in each scrambled sentence in order to create meaningful sentences. Some of sentences had more than one possible solution. However, jurors were instructed to write down only one solution and use all ten words for each sentence or as many words as they could. Jurors were given eleven minutes [20] to complete the task. The final score was calculated based on the number of errors each juror made. A single point was deducted for every mistake from the total possible score (60). If jurors did not include all words in their sentence, a single point was deducted for each missing word. A single point would also be deducted for every incorrectly positioned word.

The same trial footage, demographic/verdict questionnaire, note taking materials, and memory test used in Study 1 were also used in Study 2.

#### Procedure

The procedure was similar to that of Study 1 except that instead of handwriting measures, the participants completed the tasks in the following order: the Letter Span task, the Listening Span task, and the Word Reordering. As Study 1 (and several past studies) found no differences in the amount of trial information recalled between those who were and were not allowed to access notes during the free recall task, we removed the access to notes condition from the study design. All note taking jurors had their notes confiscated when the trial video was over. Thus, they were not able to access their notes during the memory test.

#### Coding of notes and free recall

The coding procedure of notes and free recall responses was the same as in Study 1. The inter-rater agreement between the lead author and independent raters was 98% for notes and 95% for free recall. All disagreements were resolved by the lead author and an independent reviewer who compared them and determined the correct score.

### Results

Correlational analyses were used to examine the associations between short-term memory capacity, working memory storage capacity, information processing ability, the amount of critical evidence noted down and recalled, and the amount of correct trial information noted down and recalled (see Table 2).

**Table 2:** Descriptive statistics and zero-order correlations (Pearson’s *r*) showing jurors’ short-term memory capacity, working memory capacity, information processing ability, the amount of critical evidence they noted down and recalled, and the amount of correct trial information they noted down and recalled.

Variable	Mean(±SD)	1	2	3	4	5	6
1. Short-term Memory	6.59(±1.89)						
2. Working Memory	3.29(±1.11)	.11	-				
3. Info Processing	34.06(±9.73)	.15	-.06	-			
4. Critical Notes	6.21(±2.61)	.26	-.04	.14	-		
5. Critical Recall	4.73(±1.84)	.12	.02	.04	.31*	-	
6. Correct Notes	24.41(±13.48)	.12	.09	.12	.77***	.18	-
7. Correct Recall	18.28(±7.37)	.03	.04	.19*	.21	.51***	.27*

* *p* < .05

*** *p* < .001

Three simple mediation analyses were conducted to assess (1) the direct effect of short-term memory capacity, working memory storage capacity, and information processing ability on the amount of critical evidence recalled, and (2) the indirect effect of short-term memory capacity, working memory storage capacity, and information processing ability on recall through the volume of critical evidence noted down. The models included only those jurors who took notes during the trial.

#### Short-term memory capacity

**Fig 2:**
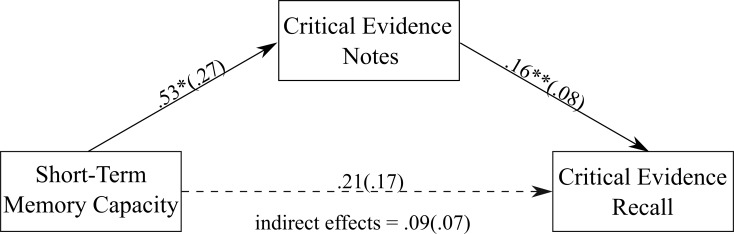
The mediation model showing the association between short-term memory capacity and the amount of critical evidence recalled, with the amount of critical evidence noted down as the mediator. Values on paths are unstandardised regression coefficients (SEs). * *p* = .05, ** *p* < .05.

A positive association was found between the amount of critical evidence jurors noted down and the amount of critical evidence recalled (see Fig 2). There was a non-significant direct effect of short-term memory capacity on the amount of critical evidence recalled, unstandardized estimate = .21 (.17), *p* = .21, 95% CI = -.12, .55. However, there was a significant indirect effect of short-term memory capacity on recall through the amount of critical evidence noted down during the trial, unstandardized estimate = .09 (0.07), 95% CI = .01, .29, κ^2^ = 0.07, *P*_*M*_ = 0.29, (κ^2^ indicates a medium effect size). More specifically, jurors with higher levels of short-term memory capacity recalled a greater amount of critical evidence, through writing down the most critical evidence during the trial.

#### Working memory storage capacity

**Fig 3:**
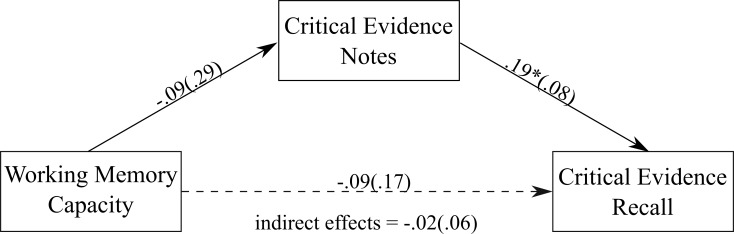
The mediation model showing the association between working memory capacity and the amount of critical evidence recalled, with the amount of critical evidence noted down as the mediator. Values on paths are unstandardised regression coefficients (SEs). * *p* < .05.

There was a positive association between the amount of critical evidence noted down and the amount of critical evidence recalled (see Fig 3). We found a non-significant direct effect of working memory storage capacity on the amount of critical evidence recalled, unstandardized estimate = 0.09 (0.17), *p* = .61, 95% CI = -.26, .43. The indirect effect of working memory storage capacity on recall, through the amount of critical evidence noted down during the trial, was also not significant, unstandardized estimate = -0.02 (0.06), 95% CI = -.18, .08, κ^2^ = 0.01, *P*_*M*_ = -0.26.

#### Information processing

**Fig 4:**
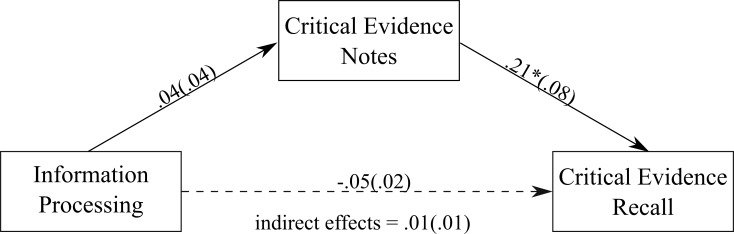
The mediation model showing the association between information processing ability and the amount of critical evidence recalled, with the amount of critical evidence noted down as the mediator. Values on paths are unstandardised regression coefficients (SEs). * *p* < .05.

We found a positive association between the amount of critical evidence noted down and the amount of critical evidence recalled (see Fig 4). There was a non-significant direct effect of information processing on the amount of critical evidence recalled, unstandardized estimate = -0.05 (0.02), *p* = .05, 95% CI = -.09, .001. Additionally, there was a non-significant indirect effect of information processing recall, through the amount of critical evidence noted down during the trial, unstandardized estimate = 0.01 (0.01), 95% CI = -.01, .04, κ^2^ = 0.06, *P*_*M*_ = 0.25.

#### Incriminating/non-incriminating evidence and verdict

As in Study 1, we conducted a logistic regression to determine whether the amount of incriminating evidence and the amount of non-incriminating evidence jurors recalled predicted their verdict (0 = not guilty, 1 = guilty). The model significantly predicted the likelihood of jurors reaching a guilty verdict, correctly identifying 77.6% of cases (χ*^2^*(2) = 31.64, Cox & Snell *R*^2^ = .31, Nagelkerke *R*^2^ = .43, *p* < .001). The amount of incriminating evidence recalled statistically predicted guilty verdicts reached by jurors, B = .72 (SE = .22), Wald = 10.96, *p* = .001; OR = 2.05, 95% CI = 1.34, 3.14, such that for every additional piece of incriminating evidence recalled, jurors were 2.05 times more likely to reach a guilty verdict. The amount of non-incriminating evidence recalled again negatively predicted jurors’ guilty verdicts, B = -.75 (SE = .23), Wald = 10.91, *p* = .001; OR = 0.47, 95% CI = 0.30, 0.74, such that for every piece of non-incriminating evidence recalled, jurors were 2.13 times less likely to reach a guilty verdict.

#### Verdict

Sixty-six per cent of jurors reached a guilty verdict: 65% in the note-taking condition and 67% in the non-note taking condition. To examine whether the condition that these jurors were in predicted their verdict, we conducted a logistic regression. As expected, and as found in Study 1, no effect was observed (χ*^2^*(1) = 0.01, Cox & Snell *R*^2^ < .01, Nagelkerke *R*^2^ < .01, *p* = .92).

#### Benefits of note taking

An independent t-test was used to examine whether note-taking jurors recalled more correct trial information than non-note taking jurors. Again we found note taking jurors recalled significantly more correct trial information (*M* = 19.72, *SD* = 7.11) than jurors who did not take notes (*M* = 15.19, *SD* = 7.07), *t*(83) = 2.75, *p* = .007 , *d* = 0.64.

### Interim discussion

Study 2 is the first to demonstrate that jurors with higher levels of short-term memory capacity recall a greater amount of critical evidence through noting down a greater amount of critical evidence during the trial. However, no significant associations were found between jurors working memory storage capacity, information processing ability and the amount of critical evidence noted down and recalled. Further, Study 2 also replicated Study 1’s finding that the type of critical evidence jurors predominantly recalled predicted their verdicts. Specifically, jurors who recalled greater amounts of incriminating evidence were more likely to find the defendant guilty; at the same time, jurors who recalled greater amounts of non-incriminating evidence were less likely to find the defendant guilty.

Furthermore, there was no association between working memory storage capacity and the amount of critical evidence noted down and recalled. This is in line with Peverly et al. [9–11] who also did not find a significant relationship between students’ working memory capacity and the amount of lecture ideas recorded in notes. Lastly, we did not find a significant association between jurors’ information processing ability and the amount of critical evidence recorded in notes and recalled. This does not support the previous findings where students’ ability to process information was associated with lecture note taking [20]. We interpret the findings in the General Discussion.

Past research indicates that attention may also be important in note taking [10]. Thus, Study 3 investigated the impact of individual differences in sustained and divided attention on juror note taking during trials and recall of trial information.

## Study 3

The aim of Study 3 was to investigate the association between jurors’ sustained and divided attention capacity, the amount of critical evidence they noted down during a trial, and the amount of critical evidence they recalled. The overload theory suggests that attentional resources become exhausted when attempting to maintain focus on the same information for extended periods of time [23,24]. Thus, presumably jurors with lower levels of sustained attention would be more likely to lose focus during the trial, causing them to take fewer notes and recall less trial information. In line with previous findings [10], we hypothesised that jurors with higher levels of sustained attention would note down more pieces of critical evidence during the trial, and that they would subsequently recall the most critical evidence.

In addition, when required to divide attention, jurors may have less time to process and encode the information because they are required to simultaneously perform two tasks [26]. Alternatively, it may be that dividing attention may reduce the number of attentional resources jurors have available for processing trial information [43]. The present study explored the association between jurors’ divided attention capacity and the amount of critical evidence noted down and recalled. Lastly, the current study also investigated the association between the type of evidence jurors predominantly recalled and their verdicts. We attempted to replicate the findings from Studies 1 and 2 which indicated that the type of critical evidence (incriminating, non-incriminating) jurors predominantly recall can predict their verdicts.

### Method

#### Participants and design

One hundred thirty-four participants (24 males, 110 females) between 18 and 59 years of age (*M* = 22.91, *SD* = 8.90) acted as mock jurors. All were eligible for jury service in England and Wales. Prior to testing, all participants provided written consent confirming they wished to take part in the study. They were a combination of undergraduate students and staff at the first author’s university and received payment in the form of either course credit or a £10 voucher. Jurors were assigned to one of three conditions: note taking with notes available during the memory test (N = 54), note taking with notes unavailable during the memory test (N = 53), and non-note taking (N = 27). Participants were assigned to conditions as in Studies 1 and 2. The study was approved by the Health and Life Sciences Committee on Research Ethics of the University of Liverpool.

#### Stimuli

The Lottery subtest of the Test of Everyday Attention [44] was used to measure sustained attention, which is the ability to maintain attention to a constant and boring task. Jurors listened to a pre-recorded 10-min audio file containing strings of numbers and letters which represented ‘lottery tickets’ (e.g., BC143). Whilst doing this, they were instructed to listen out for winning tickets. The winning tickets were those that ended in 55. Upon hearing these numbers, they were asked to immediately write down the two letters preceding them. Jurors received one point for every correct set of letters written down and could achieve a maximum score of 12 points.

The Dual-Task technique pioneered by Baddeley and Hitch [15] is commonly used to measure the impact of dividing attention during encoding on subsequent memory performance [26,28,45] and assess attentional resources devoted to text production [13]. The task was designed in PsychoPy [46] and included two parts: a Single Reaction Time (RT) task and a Dual RT task. During the Single RT task, jurors listened to 30 auditory beeps distributed randomly at intervals with a mean of 10 seconds (range 5–15 seconds). Jurors were instructed to respond to each beep as fast as possible by pressing the spacebar with their non-dominant hand. Their response time was recorded. Jurors’ baseline RT was calculated by computing the mean score for the last 25 responses (the first five responses were considered practice trials). The Dual RT Task required jurors to simultaneously perform two tasks: the RT task and a writing task. In the writing task, all jurors were instructed to compose an essay on the pros and cons of a proposed increase in student tuitions fees. They were provided with an A4 notebook and a pen. If jurors finished their first essay before the RT task was over, they were instructed to immediately start writing a second essay which involved describing their favourite book or film. Jurors were told to concentrate fully on writing the essay but to try to respond to the beeps as rapidly as possible by pressing the spacebar with their non-dominant hand. The beeps were distributed randomly at intervals with a mean of 30 seconds (range 15–45 seconds). The Dual RT task provided a measure of jurors’ RT during divided attention, which was calculated by computing the mean RT score for the last 25 responses.

During both RT tasks, jurors positioned their non-dominant hand on top of a sketch of a hand positioned in front of the keyboard. The researcher ensured that the sketch was correctly positioned to ensure that all jurors’ hands were at an equivalent distance to the keyboard on all trials (so RTs would not be influenced by hand position). The interference in RT was calculated by subtracting the baseline RT from the Dual RT. This provided the final divided attention score which is a measure of cognitive effort devoted to writing while listening. Both RT tasks lasted approximately 20 minutes.

The same trial footage, demographic/verdict questionnaire, note taking materials, and memory test used in Studies 1 and 2 were also used in Study 3.

#### Procedure

The procedure was similar to that of Studies 1 and 2, except that jurors completed the Lottery subtest of the Test of Everyday Attention and the Dual-Task before they watched the trial video.

#### Coding of notes and free recall

The coding procedure for the notes and free recall responses was the same as in Study 1. The inter-rater agreement between the lead author and independent raters was 83% for notes and 81% for free recall. All disagreements were resolved by the lead author and an independent reviewer who compared them and determined the correct score.

### Results

Correlational analyses were used to examine the associations between sustained attention, divided attention, critical evidence noted down and recalled, and correct trial information noted down and recalled (see Table 3).

**Table 3:** Descriptive statistics and zero-order correlations (Pearson’s *r*) showing jurors’ sustained attention capacity, divided attention capacity, the amount of critical evidence they noted down and recalled, and the overall amount of correct trial information they noted down and recalled.

Variable	Mean(±SD)	1	2	3	4	5
1. Sustained Attention	9.36(±1.71)	-				
2. Divided Attention	0.23(±0.17)	-.04				
3. Critical Notes	6.76(±2.84)	.26**	-.13	-		
4. Critical Recall	6.39(±2.16)	.19*	.21*	.52***	-	
5. Correct Notes	26.21(±13.59)	.24*	-.12	.76***	.39***	-
6. Correct Recall	22.87(±9.48)	.09	-.06	.41***	.67***	.49***

* *p* < .05

** *p* < .01

*** *p* < .001

Two simple mediation analyses were conducted to assess (1) the direct effect of jurors’ sustained and divided attention capacity on the amount of critical evidence recalled, and (2) the indirect effect of sustained and divided attention capacity on recall through the amount of critical evidence noted down. The models included only those jurors who took notes during the trial.

#### Sustained attention

**Fig 5:**
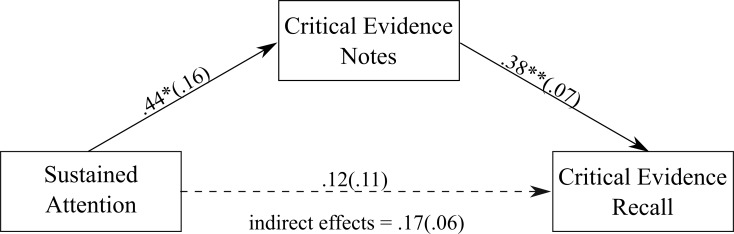
The mediation model showing the association between sustained attention and the amount of critical evidence recalled, with the amount of critical evidence noted down as the mediator. Values on paths are unstandardised regression coefficients (SEs). * *p* < .05; ** *p* < .001.

Jurors with higher sustained attention capacity noted down a greater amount of critical evidence. There was also a positive association between the amount of critical evidence jurors noted down and the amount of critical evidence they recalled. Fig 5 shows a non-significant direct effect of sustained attention on the amount of critical evidence recalled, unstandardised estimate = .12 (SE = .11), 95% CI = -.10, .35. The indirect effect was significant, with jurors who had a higher sustained attention capacity recalling a greater amount of critical evidence via the amount of critical evidence noted down during the trial, unstandardised estimate = .17 (SE = .06), 95% CI = .06, .32, κ^2^ = 0.14, *P*_*M*_ = 0.57 (κ^2^ indicates a medium effect size).

#### Divided attention

**Fig 6:**
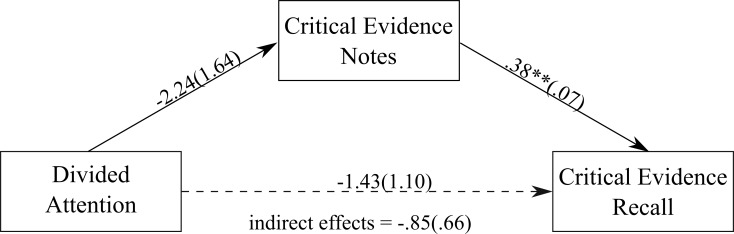
The mediation model showing the association between divided attention and the amount of critical evidence recalled, with the amount of critical evidence noted down as the mediator. Values on paths are unstandardised regression coefficients (SEs). ** *p* < .001.

Divided attention capacity was not associated with the amount of critical evidence noted down during the trial, which was unexpected. However, as above, jurors who noted down a greater amount of critical evidence recalled a greater amount of critical evidence. Fig 6 shows a non-significant direct effect of divided attention on the amount of critical evidence recalled, unstandardised estimate = -1.43 (SE = 1.10), 95% CI = -3.60, .75. Unlike the sustained attention model, no significant indirect effect of the volume of critical evidence noted down during the trial was found, unstandardised estimate = -.85 (SE = .66), 95% CI = -2.49, .13, κ^2^ = 0.07, *P*_*M*_ = 0.37.

#### Incriminating/non-incriminating evidence and verdict

We conducted a logistic regression to assess whether the amount of incriminating evidence and the amount of non-incriminating evidence jurors recalled predicted their verdict (0 = not guilty, 1 = guilty), as in the Studies 1 and 2. The overall model significantly predicted the likelihood of jurors reaching a guilty verdict, correctly identifying 67.2% of cases (χ*^2^*(2) = 29.86, Cox & Snell *R*^2^ = .20, Nagelkerke *R*^2^ = .27, *p* < .001). The amount of incriminating evidence recalled statistically and positively predicted the likelihood of guilty verdicts being reached by jurors, B = .44 (SE = .14), Wald = 9.24, *p* = .002; OR = 1.55, 95% CI = 1.17, 2.04, such that for every additional piece of incriminating evidence recalled, jurors were 1.55 times more likely to reach a guilty verdict. Further, the amount of non-incriminating evidence recalled negatively predicted the likelihood of guilty verdicts, B = -.48 (SE = .13), Wald = 14.71, *p* < .001; OR = 0.62, 95% CI = 0.48, 0.79, such that for every piece of non-incriminating evidence recalled, jurors were 1.61 times less likely to reach a guilty verdict.

#### Verdict

Fifty-three per cent of jurors reached a guilty verdict: 41% in the access to notes during retrieval condition, 60% in the no access to notes during retrieval condition and 63% in the non-note taking condition. A logistic regression was conducted to test whether the condition predicted verdicts. In line with our previous findings, no effect was observed (χ*^2^*(2) = 5.52, Cox & Snell *R*^2^ = .04, Nagelkerke *R*^2^ = .05, *p* = .06).

#### Benefits of note taking and note access at retrieval

An independent t-test was carried out to examine whether jurors who took notes during the trial recalled more correct trial information than those who did not take notes. Note taking jurors recalled significantly more correct trial information (*M* = 24.35, *SD* = 9.54) than non-note taking jurors (*M* = 17.04, *SD* = 6.61), *t*(132) = 3.75, *p* < .001 , *d* = 0.88.

An independent t-test was used to examine whether jurors who were able to access their notes during the memory test recalled more information than those who were not permitted to access their notes. Consistent with Study 1, there was no significant difference in the amount of information recalled by note takers who could not access their notes during the memory test (*M* = 26.24, *SD* = 13.23), and those who had access to their notes during the memory test (*M* = 26.17, *SD* = 14.08), *t*(105) = .03, *p* = .98, *d* = 0.01.

### Interim discussion

Study 3 demonstrates that jurors with higher levels of sustained attention recall a greater amount of critical evidence, and this is via noting down a greater amount of critical evidence during the trial. This finding is in line with previous research showing that students who have higher levels of sustained attention capacity take the most notes and recall the most lecture material [10]. Our study extends this finding to a new domain (i.e. jurors). In an exploratory analysis, Study 3 found jurors’ divided attention capacity was not related to the amount of critical evidence they noted down during the trial. These findings are considered further in the General Discussion.

We found that note taking jurors who recalled greater amounts of incriminating evidence were more likely to reach a guilty verdict. Also, jurors who recalled more non-incriminating evidence were less likely to reach a guilty verdict. This study replicates the findings from Studies 1 and 2 which further supports the idea that jurors’ verdicts are predicted by the amount of trial evidence they recall.

## General discussion

Note taking during trials improves jurors’ recall of trial evidence, regardless of whether they are able to access their notes during the memory test. Here, three studies aimed to test whether individual differences that have been related to effective note taking within the educational psychology literature might predict the volume of critical evidence jurors note down during a trial and whether this, in turn, predicts the amount of critical evidence they can later recall. We also tested whether the type of evidence jurors predominantly recalled predicted their verdicts. We found that handwriting speed, short-term memory capacity, and sustained attention capacity had an indirect effect on the amount of critical evidence jurors recalled through the amount of critical evidence they noted down. In other words, jurors with faster handwriting speed, higher levels of short-term memory capacity and sustained attention capacity recollected a greater amount of critical evidence through noting down a greater amount of critical evidence during the trial. However, working memory storage capacity, information processing, and divided attention were not found to be associated with either measure. Of key importance to the judicial process, the three studies consistently found that verdicts were predicted by the amount of critical incriminating and non-incriminating trial evidence jurors recalled. Specifically, we found that jurors who recalled more incriminating evidence were more likely to find the defendant guilty, and those who recalled more non-incriminating evidence were less likely to find the defendant guilty. The studies are the first to demonstrate that the type of critical evidence jurors predominantly recall predicts the verdicts they reach. Thus, in order to make informed and just decisions, jurors must remember as much of the critical evidence as possible. This is affected how many notes jurors are able to take during a trial.

Study 1 found that jurors with faster handwriting speed recalled greater amounts of critical evidence, and this effect was mediated by the amount of critical evidence they noted down during the trial. Our findings are in line with findings from educational psychology, where the quality of notes mediated the association between handwriting speed and recall of lecture material [11]. We extend this finding to a new domain. Note takers with slower handwriting speeds may find it more difficult to note down the information before it is forgotten, which places a heavy cognitive load on their working memory [12,13]. Thus, Study 2 investigated the role of working memory in juror note taking and recall. In addition, it examined the role of short-term memory capacity and information processing ability.

Study 2 found that jurors with higher levels of short-term memory capacity recalled a greater amount of critical evidence, and this was via a greater amount of critical evidence noted down. However, no associations were found between jurors’ working memory capacity, information processing ability and note taking or recall of critical evidence. We presumed that jurors with a higher working memory storage capacity would take more notes as they could store larger amounts of trial information in memory whilst focusing on making notes. However, this was not supported by the current findings.

We expected both short-term memory capacity and working memory capacity to be associated with note taking. Whilst jurors are note taking, both their short-term memory and working memory capacity are fully engaged with the current information. As such, those with lower capacity may be unable to store and process the incoming information in memory and they subsequently fail to encode it/note it down. Thus, we would expect an adequate measure of short-term memory capacity to be associated with note taking, as found by the present study. However, presumably working memory capacity should be a better predictor of note taking during trials as it captures more accurately the complexity of the processes involved in note taking (i.e. storing, processing, manipulating incoming information). Therefore, it is surprising that this association was not found in the present study.

It is possible that the working memory task we used (listening span) does not accurately measure the demands placed on working memory by note taking. This is the most likely explanation for the lack of significant associations between working memory capacity and notes demonstrated by the present and previous studies [9,11]. Working memory has previously been found to be associated with the number of themes in lecture notes [40] and test performance [47]. However, previous studies used different working memory tasks (i.e. a reading span task and a combined score of three memory span tasks) to the one used in the present study, suggesting that the association may be sensitive to the type of working memory task employed. Further research using a battery of working memory tasks is needed to clarify the association between working memory and note taking. Furthermore, others have found a significant association between lecture note taking and working memory when students were asked to organise their notes but not when they were asked to record everything that was said [19]. This suggests that note taking strategies may affect the association between working memory and note taking [14]. Whilst we did not examine jurors’ note taking strategies, it may be that working memory capacity was not significantly associated with note taking in the present study as we did not ask jurors to organise their notes and they were simply writing everything down (in a pure transcription style).

The lack of association found between jurors’ information processing ability, notes, and recall contradicts previous findings [20]. However, in the present study notes were assessed for the volume of critical evidence whereas Kiewra and Benton [20] assessed notes for complex propositions and main ideas. Thus, it may be that the information processing ability is related to processing more complex information. Future research should investigate the association between processing complex information and note taking.

Study 3 showed that jurors with higher levels of sustained attention recalled more critical evidence, which was via noting down more critical evidence while they watched the trial. This supports previous findings [10] and extends them to a new domain. These findings can potentially be interpreted in terms of the overload theory, which suggests individuals’ attentional resources can become exhausted when attempting to maintain focus on the same information (e.g., trial evidence) for extended periods of time [23,24]. Thus, jurors with lower levels of sustained attention may have the greatest difficulty maintaining focus on a trial, causing them to take fewer notes and recall less trial evidence.

We did not find a significant association between jurors’ divided attention capacity and the amount of critical evidence they noted down during the trial or their subsequent recall of that evidence. The reason why sustained attention predicted jurors’ note taking and recall but divided attention did not is unclear. One possible explanation is that the act of note taking focuses jurors’ attention on the trial so that their attention is not truly divided, and thus note taking/recall are subsequently unaffected. Alternatively, this null result may be a consequence of the sample used here. The undergraduate students who took part in our studies would have had experience of note taking during presentations (i.e., in their lectures). Research suggests that individuals who have extensive practice at simultaneously reading stories and writing dictated words are less affected by the demands of dividing attention between these tasks [48]. It may be the case that our student jurors’ prior experience of note taking during lectures meant the cognitive demands of note taking were less than would be found in a population with little or no experience. Future research could examine this issue by testing jurors who are novice note takers and compare them to more experienced note takers.

The present findings showed no difference in recall between jurors who were able to access their notes during the memory test and those who did not have access to their notes. This is in line with prior research [7,8]. It may be that note taking encourages generative processing of the new information [49–51] which leads to deeper encoding of such information [52–54]. In addition, generative processing involves actively creating connections between diverse parts of new information (or between the new information and one’s own prior knowledge) so that it is stored in memory in a meaningful and organised way [55,56]. Subsequently, note takers may find it easier to recollect the newly learned information as recalling one piece of information cues the recall of other related information [57,58]. Thus, the evidence suggests that the act of note taking plays a central role in enhancing jurors’ recall of trial information.

### Limitations

One limitation of the present study, which poses a threat to the ecological validity of the findings, is that the trial lasted only 30 minutes. Real trials can last days or weeks. Note taking over longer periods of time may impose more cognitive demands on jurors’ attention (e.g., those with higher levels of sustained attention capacity may gradually lose focus). Additionally, it may impose more physical demands on jurors’ handwriting speed (e.g., those with faster handwriting speed may slow down after extended periods of time due to tiredness). Note taking behaviours over extended periods of time have not yet been investigated. Nevertheless, we did find significant indirect effects of individual differences and these may be even more evident in real and longer trials. In addition, the constraints of laboratory settings affect all laboratory-based juror studies [59]. Of importance, we opted to use a trial video rather than a brief trial transcript (as used by others) based on suggestions to increase the ecological validity of laboratory-based juror studies [60]. Although the majority of the participants were undergraduate students, all were jury eligible in the UK so could potentially be selected for jury service. Of importance, evidence suggests there is no difference with regards to verdicts reached between student and non-student samples in mock jury research [59,61].

### Future directions

The present studies assessed the effects of note taking on individual jurors’ recall of trial evidence. However, future studies should aim to examine the effects of note taking on deliberations, and the effects of deliberation on recall of trial evidence. It is important to assess whether note taking facilitates or impairs deliberation. It should also be assessed whether deliberation facilitates or impairs jurors’ recall of trial evidence. Addition, it would be interesting to examine the potential impact of biases (which may be caused by pre-trial publicity) on note taking and the recall of trial information. Jurors who have been exposed to pre-trial publicity may selectively note down the evidence that is consistent with their biases. Future studies should also consider using a trial video with a less ambiguous outcome (i.e. there is a wealth of evidence suggesting that the defendant is guilty or not guilty) to examine whether the findings from the present study can be replicated in these circumstances. Future researchers could also examine whether informing participants that they will be able to access their notes during the memory test will have an impact on their note taking strategies. The current studies did not measure whether jurors used or looked at their notes during the memory test, and thus future research should consider investigating this and its impact upon recall. In addition, since our studies tested the various individual differences in isolation, researchers are encouraged to study all of those factors together in a single study in order to assess any potential covariance among the individual differences. Lastly, future research should also examine whether other individual differences may influence juror note taking and recall, for instance prior experience of note taking, language comprehension, and age.

### Implications

The findings from the present study have an important applied value. Although note taking has been found to improve recall of trial information, some judicial systems deny jurors the opportunity to take notes during trials. The present studies show that note taking increases recall of critical evidence. When jurors remember more trial information they presumably have a greater chance of reaching a just verdict. Based on our findings, all jurors should be permitted to take notes during trials. Furthermore, the present studies show that jurors with faster handwriting speeds, higher short-term memory capacity, and higher sustained attention capacity benefit from note taking by recalling greater amounts of critical evidence. Since we identified handwriting speed as a feature of note taking that affects recall, it may be that providing instructions for speeded writing may help potential jurors note down more trial information and subsequently reach more informed verdicts. In addition, the current findings demonstrated that sustained attention was associated with how much critical evidence jurors recalled. Currently, jurors in the US are often required to attend court sessions of no less than 80 minutes after which they are allowed a 15-minute break. Thus, note-taking jurors with lower sustained attention levels may benefit from taking more regular breaks to ensure that they stay focused on the trial evidence and to prevent them from missing critical evidence which could then impact upon their decision making.

Additionally, the current findings have an important theoretical value. Little was known about individual differences in note taking aside from a small number of studies from the educational psychology literature. We demonstrate that the impact of handwriting speed and sustained attention on note taking and recall generalise to another domain. In addition, we identify that short-term memory capacity is also an important factor that is associated with note taking and recall.

### Conclusion

The present studies demonstrated that jurors with faster handwriting speed, higher short-term memory capacity, and higher sustained attention recalled greater amounts of critical evidence, and this effect was mediated by the amount of critical evidence they noted down during the trial. However, working memory storage capacity, information processing ability, and divided attention were not found to be significantly associated with note taking and recall. In addition, all studies found that the type of evidence jurors recalled predicted their verdicts. The current findings have an important theoretical value, such that they identify individual differences that can predict the positive association between juror note taking and recall. This is particularly important given that jurors who recalled more incriminating evidence were more likely to reach a guilty verdict, and jurors who recalled more non-incriminating evidence were less likely to find the defendant guilty.

## Supporting information

S1 Dataset Data files for each of the studies. (ZIP)
